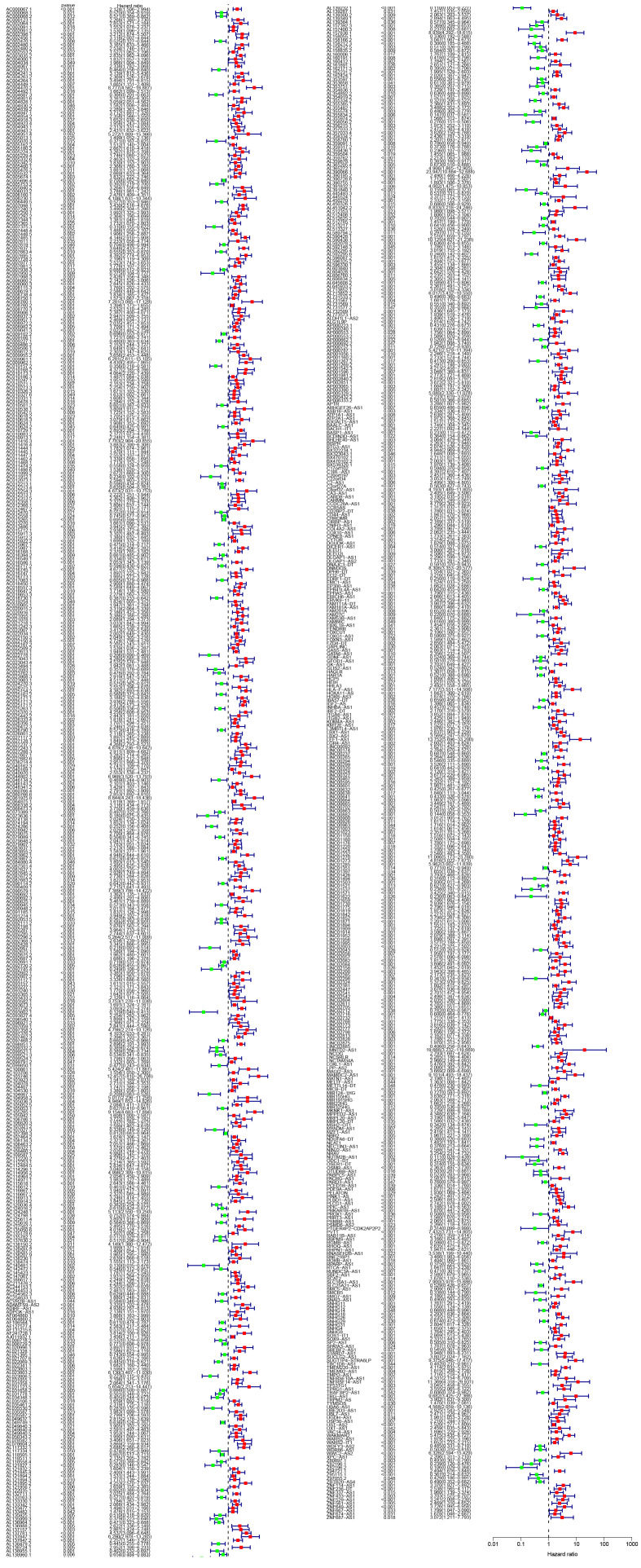# A disulfidptosis-associated long noncoding RNA signature to predict low-grade glioma classification, prognosis, tumor microenvironment, and therapy regimens: Observational study

**DOI:** 10.1097/MD.0000000000039316

**Published:** 2024-08-23

**Authors:** Xiaohong Qin, Zhibiao Chen, Liquan Wu, Rui Ding

**Affiliations:** a Department of Neurosurgery, Renmin Hospital of Wuhan University, Wuhan, Hubei, China.

**Keywords:** disulfidptosis, LncRNA, low-grade glioma, prognosis, tumor microenvironment

## Abstract

This study aimed to investigate the function of disulfidptosis-associated long noncoding RNAs (DAlncRNAs) in low-grade gliomas (LGG) through bioinformatics analysis and construct a signature to predict the classification, prognosis, tumor microenvironment, and selection of immunotherapy and chemotherapy in LGG. Genomic, clinical, and mutational information of 526 patients with LGG was retrieved from The Cancer Genome Atlas repository. A nonnegative matrix factorization algorithm was applied to classify patients with LGG. Univariate, LASSO regression, and multivariate Cox regression analyses were performed to determine prognostic DAlncRNAs. Following the median risk score, we defined the sample as a high-risk (HR) or low-risk group. Finally, survival, receiver operating characteristic curve, risk curve, principal component, independent prognosis, risk difference, functional enrichment, tumor microenvironment, immune cell infiltration, mutation, and drug sensitivity analyses were performed. Patients were classified into C1 and C2 subtypes associated with disulfidptosis. Eight prognostic DAlncRNAs (AC003035.2, AC010157.2, AC010273.3, AC011444.3, AC092667.1, AL450270.1, AL645608.2, and LINC01571) were identified, and a prognostic signature of LGG was developed. The DAlncRNA-based signature was found to be an independent prognostic factor in patients with LGG, thereby constructing a nomogram. In addition, in the HR group, immune function was more active and the tumor mutation burden was higher. The patients were mainly composed of subtype C2, and their prognosis was worse. Immunotherapy and chemotherapy were predicted in the HR and low-risk groups, respectively. Our study, based on DAlncRNAs, highlights 2 disulfidptosis-associated LGG subtypes with different prognostic and immune characteristics and creates a novel disulfidptosis-associated prognostic signature, which may inform the classification, prognosis, molecular pathogenesis, and therapeutic strategies for patients with LGG.

## 1. Introduction

Gliomas, poor prognoses, and high relapse rates are the most common intracranial tumors. Diffuse low-grade and intermediate-grade gliomas (grades II and III) collectively constitute low-grade gliomas (LGG), which are also subdivided into isocitrate dehydrogenase (IDH) mutated, 1p/19-deficient gliomas or oligodendrogliomas, IDH-mutated, 1p/19q-retained, p53-mutated, ATRX-mutated gliomas, or astrocytomas.^[1,2]^ Although LGG has a better prognosis than high-grade glioma through surgical resection, chemotherapy, and radiotherapy, it continues to grow at a rate of 4 to 5 mm per year, and more than half of LGG patients will eventually progress to highly invasive gliomas.^[3,4]^ Therefore, it is imperative to excavate neoteric biomarkers for LGG therapy.

Currently, although systemic treatment regimens for immunotherapy in solid tumors (e.g., renal cancer,^[5]^ breast cancer,^[6]^ etc) are being progressed and optimized, and several potentially predictive biomarkers such as the expression level of programmed cell death 1 ligand 1,^[5]^ immortal time bias,^[7]^ and Eastern Cooperative Oncology Group performance status^[8]^ are being evaluated, credible markers for the selection of patients that may benefit from immunotherapy and for guiding the therapeutic strategy remain far from being validated. Recently, Liu et al identified a novel cell death mechanism, disulfidptosis, in SLC7A11^high^ UMRC6 cells during glucose starvation. A large buildup of disulfide bonds (S–S) induces anomalous S–S cross-linking between actin cytoskeletal proteins and cytoskeletal contraction, which disrupts their tissues and culminates in the collapse of the actin network and cell death.^[9]^ This study opens a new approach to anticancer therapy that targets the pathophysiological effects of disulfide stress. Intriguingly, a plethora of bioinformatics studies have shown that disulfidptosis regulators are linked to the prognosis of bladder cancer,^[10]^ lung adenocarcinoma,^[11]^ and hepatocellular carcinoma.^[12]^

In the past few years, long noncoding RNAs (lncRNAs) have become biomarkers of considerable interest. Alterations in lncRNA expression and concomitant mutations conduce to tumorigenesis and metastasis, thereby exacerbating carcinogenesis.^[13]^ Previous studies have shown that lncRNAs are tightly correlated with hyperplasia, assault, and glioma cell outcome.^[14,15]^ However, there are few studies on the relationship between disulfidptosis-associated long noncoding RNAs (DAlncRNAs) and LGG.

Until now, the impact of DAlncRNAs on LGG classification, prognosis, immune microenvironment, and therapy is ill-defined. Thus, this study probed the function of DAlncRNAs in LGG by bioinformatics analysis and constructed a signature to predict the classification, prognosis, and tumor microenvironment, as well as the selection of immunotherapy and chemotherapy for LGG.

## 2. Materials and methods

### 2.1. Data acquisition and processing

The genomic, clinical, and mutational information (survival time, sex, age, and WHO grade) of 526 LGG patients was retrieved from The Cancer Genome Atlas (TCGA, https://portal.gdc.cancer.gov/Repository) repository. The data were standardized using the “SVA” R package. Mutational information was processed by the “maftools” R package. Twenty-four disulfidptosis-associated genes (DAGs) were obtained from the publications of Liu, including ACTB, CAPZB, FLNA, NUBPL, CD2AP, ACTN4, OXSM, DSTN, NCKAP1, FLNB, GYS1, INF2, RPN1, IQGAP1, LRPPRC, MYH10, NDUFS1, MYH9, SLC3A2, MYL6, NDUFA11, PDLIM1, SLC7A11, and TLN1.^[9]^ The lncRNAs associated with the disulfidptosis genes were acquired utilizing correlation analysis in the “limma” R package.

### 2.2. Nonnegative matrix factorization (NMF) clustering identification of LGG patients

To reduce the dimensions of NMF clustering, univariate Cox regression analysis was adopted to ascertain lncRNAs with prognostic significance. Only prognostic lncRNAs closely related to disulfidptosis were retained as dimensions and parameters of NMF clustering. The “NMF” packet was applied to cluster the LGG samples, and the clustering number was set to 2 to 10. The criterion “brunet” selection was checked, and conducted 10 loops. We ascertained the optimal clustering values for the cophenetic, dispersion, evar, rss, and silhouette indicators. The “pheatmap” packet was applied to obtain the typing results. The overall survival (OS) and progression-free survival (PFS) of the various subtypes were analyzed using the “survival” R package, and Kaplan–Meier survival curves (K-MSC) were mapped. “MCPcounter” quantified the absolute abundance of 10 cell types.^[16]^ The “ggalluvial” packet was selected for the Sankey mapping.

### 2.3. Construction and validation of a DAlncRNAs signature

We modeled the prognosis of disulfidptosis to appraise the relationship between disulfidptosis and LGG. The 513 LGG samples with comprehensive clinical data were randomly divided into a training set (TS, n = 257) and validation set (VS, n = 256) at a 1:1 ratio. First, the DAlncRNAs obtained from co-expression analyses were subjected to univariate Cox analysis in the TS to identify the significant prognostic DAlncRNAs. We further selected lncRNAs using the LASSO Cox regression analysis to refine the signature. Finally, multivariate Cox analysis was applied to further select prognostic DAlncRNAs in TS to construct a prognostic signature. $Risk\;socre=\overset{n}{\underset{i=1}{\sum }}\left(expi*coef\;i\right)$ (exp_i_: expression value of each lncRNA; coef_i_: survival correlation regression coefficient of each lncRNA).^[17]^ Following the median risk score of TS, we defined all samples (AS), TS, and VS as high-risk (HR) and low-risk (LR) groups. Survival analysis was carried out in the AS, TS, and VS, and receiver operating characteristic (ROC) curves, risk curves, and principal component analysis (PCA) were used to verify the credibility of the signature prediction.

### 2.4. Establishment and verification of the nomogram

By combining the results of the 2 regression analyses, a nomogram of the DRlncRNA risk score and clinical characteristics was established. The R package “rms” was used to draw a calibration plot to test prediction accuracy. The “timeROC” packet was also employed to evaluate the area under the curve (AUC) of ROC to examine the prognostic ability of the nomogram.

### 2.5. Functional enrichment analysis

Gene Ontology (GO) and Kyoto Encyclopedia of Genes and Genomes (KEGG) enrichment analyses were carried out applying the “clusterProfiler” packet to investigate the significant functions and pathways of risk differentially expressed genes (RDEGs).^[18]^ The reference gene set files “c5.go.Hs.symbols.gmt” and “c2.cp.kegg.Hs. symbols.gmt” were used for gene set enrichment analysis (GSEA) to look into the differences in functions and pathways between the HR and LR teams. GO, KEGG, and GSEA results were visualized by the “ggplot2” packet.

### 2.6. Analysis of the immunologic microenvironment of LGG cells

The ratio of immune-related cells in 2 groups was calculated using the R packet “Estimate.” The “CIBERSORTx” packet was applied to compute the enrichment of multiple immune cells in LGG.^[19]^ The enriched scores of various immune cell subgroups were quantified by single-gene set enrichment analysis to study the relevance of the risk score to immune cells and their function.

### 2.7. Tumor mutation burden (TMB) analysis in LR and HR groups

TMB is the sum of mutations per million bases in the tumor tissue and is counted as the total number of nonsynonymous variants in all samples, including deletions, splice sites, nonsense mutations, missense mutations, and insertions.^[20]^ The R packet “mafTools” was applied to present the mutated genes and their proportions in the different risk groups. A violin diagram was applied to demonstrate between-group differences in TMB. Next, patients with LGG were classified into a high TMB (H-TMB) team and a low TMB (L-TMB) team, taking into account the median TMB score.

### 2.8. Tumor immune dysfunction and exclusion (TIDE) and drug sensitivity analysis

The TIDE score was used to evaluate the ability of LGG immune evasion to predict the immune checkpoint blocking response.^[21]^ Drug expression and sensitivity data were accessed from the Genetics of Drug Sensitivity in Cancer (https://www.cancerrxgene.org/). Utilizing the “OnCopredict” packet to compute the drug sensitivity across the HR and LR teams.

### 2.9. Statistical analysis

Statistical analyses were performed using R version 4.2.3. Univariate, LASSO and multivariate Cox regression analyses were employed to determine the independent prognostic factors. Dichotomous data were tested using the chi-squared test. The *t*-test was applied to normally distributed variables and the Wilcoxon test was applied to non-normally distributed variables.

## 3. Results

### 3.1. NMF algorithm divided LGG patients into 2 subtypes

**Figure 1.**
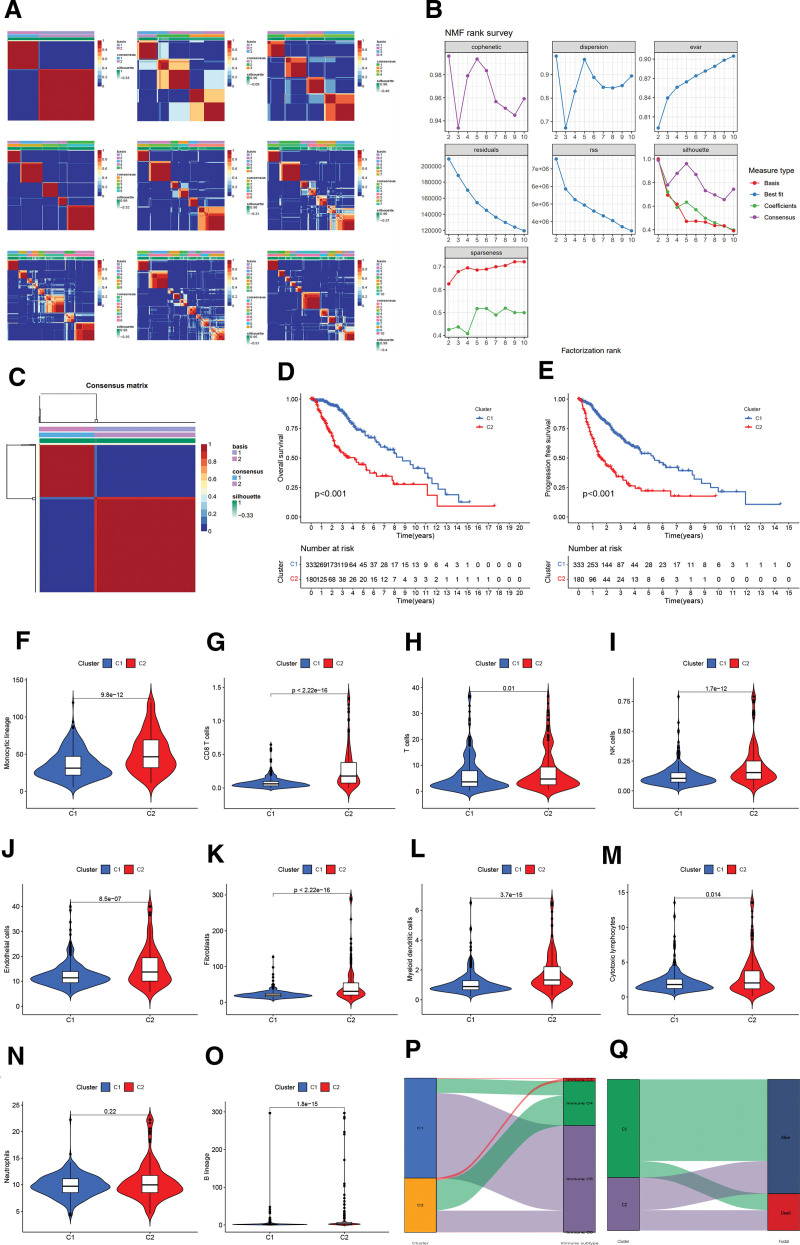
The subgroups of LGG patients were screened by NMF clustering, and the differences in immune cells between subgroups were analyzed. (A) Consensus map of NMF clustering. (B) Rank survey of NMF clustering. (C) The NMF algorithm determined the best clustering value as 2; (D and E) OS and PFS of both subtypes. (F–O) Violin diagrams of immune cell difference analysis between the 2 subtypes. (P) Sankey diagram of the corresponding relationship between 2 immunophenotypes and previous immunophenotypes of pan-cancer. (Q) Sankey diagram of the corresponding links among the 2 immunophenotypes and the survival status of LGG patients. LGG = low-grade glioma, NMF = non-negative matrix factorization, OS = overall survival, PFS = progression-free survival.

First, we used the NMF algorithm to cluster LGG samples concerning the expression of DAlncRNAs. Optimal cluster number 2 (C1 and C2) was determined (Fig. 1A–C). The K-MSC indicated that the samples in C1 had better OS and PFS (Fig. 1D and E). Subsequently, a difference analysis of the immune cells in the 2 subtypes was conducted.

The results showed a predominance of monocytes, CD8 T cells, T cells, NK cells, endothelial cells, fibroblasts, myeloid dendritic cells, cytotoxic lymphocytes, and B lineage cells in C2 patients, whereas there was no difference in neutrophils between the 2 subtypes (Fig. 1F–O).

Next, we drew a Sankey diagram to compare the correlation between the 2 immunophenotypes and the previous pan-cancer immunophenotype. The results showed that except for C6, which belongs to type C2, there was no obvious corresponding relationship between the other immunophenotypes and our immunophenotypic results (Fig. 1P). At the same time, we also used a Sankey diagram to show the corresponding relationship between the 2 types and the survival status of patients. The results showed that most of the patients in C1 were alive, and the dead patients were mainly from C2 (Fig. 1Q).

### 3.2. Eight prognostic DAlncRNAs were ascertained, and a prognostic signature of LGG was developed

**Figure 2.**
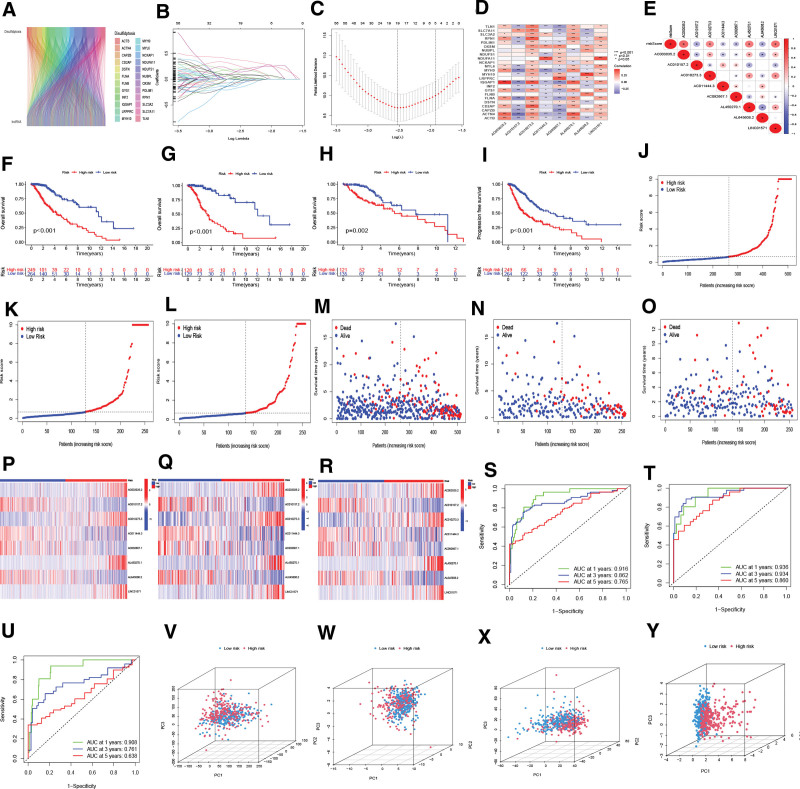
Identification and validation of DAlncRNAs with prognostic significance and modeling of risk scores. (A) The Sankey diagram shows the links between lncRNAs and 24 disulfidptosis-related mRNAs. (B and C) Lasso regression analysis screened DAlncRNAs. (D) The co-expression relationship between the 8 DAlncRNAs and 24 disulfidptosis-related mRNAs. (E) Co-expression analysis of 8 DAlncRNAs and risk scores. (F–H) K-MSC of OS of the HR and LR groups in the AS, TS, and VS. (I) K-MSC of PFS of the HR and LR in the TS. (J–L) Risk curves in the AS, TS, and VS, respectively. (M–O) Survival state diagrams in the AS, TS, and VS. (P–R) Risk heatmap distributed in the AS, TS, and VS. (S–U) ROC curves in the AS, TS, and VS. (V–Y) PCA based on all genes (V), DAGs (W), all DAlncRNAs (X), and 8 DAlncRNAs (Y). AS = all samples, DAGs = disulfidptosis-associated genes, DAlncRNAs = disulfidptosis-associated long non-coding RNA, LR = low-risk, PCA = principal component analysis, ROC = receiver operating characteristic, TS = training set, VS = validation set.

Pearson correlation analysis was carried out according to the standard of |R|>0.4 and *P* < .001, and we extracted co-expression information from 513 LGG patients and obtained 3627 prognostically relevant DAlncRNAs (Table S1, Supplemental Digital Content, http://links.lww.com/MD/N403). The correlations among the 24 mRNAs and 3627 DAlncRNAs are indicated in the Sankey diagram (Fig. 2A). In addition, 513 LGG samples were randomly defined as TS (n = 257) and VS (n = 256) at a 1:1 ratio (Table S2, Supplemental Digital Content, http://links.lww.com/MD/N404). Chi-square analysis showed that all *P* > .05, demonstrating that there was no difference in clinical traits between TS and VS (Table S3, Supplemental Digital Content, http://links.lww.com/MD/N405). We obtained 855 prognostic DAlncRNAs in TS using univariate regression analysis (*P* < .05) (Fig. S1, Supplemental Digital Content, http://links.lww.com/MD/N402). Subsequently, we carried out LASSO Cox regression analysis of the 855 DAlncRNAs associated with prognosis in TS, resulting in 19 lncRNAs (Fig. 2B and C). Multivariate Cox analysis further identified 8 prognostic DAlncRNAs in TS, including AC003035.2, AC010157.2, AC010273.3, AC011444.3, AC092667.1, AL450270.1, AL645608.2, and LINC01571 (Table S4, Supplemental Digital Content, http://links.lww.com/MD/N406). The co-expression links between the 8 DAlncRNAs and 24 disulfidptosis-associated mRNAs are displayed in Figure 2D. A prognostic risk model composed of 8 DAlncRNAs was developed. Risk score of each sample = (0.5590 × AC003035.2 exp_i_) + (‐0.7938 × AC010157.2 exp_i_) + (0.6228 × AC010273.3 exp_i_) + (‐0.3397 × AC011444.3 exp_i_) + (‐0.9616 × AC092667.1 exp_i_) + (0.2945 × AL450270.1 exp_i_) + (‐0.4732 × AL645608.2 exp_i_) + (0.6249 × LINC01571 exp_i_). The co-expression relationship between the 8 DAlncRNAs and risk score is shown in Figure 2E. Following the median risk score, the samples in the TS group were classified into the HR (n = 128) and LR (n = 129) teams (Table S5, Supplemental Digital Content, http://links.lww.com/MD/N407). According to the median risk score in TS, VS was sorted into an HR team (n = 121) and an LR team (n = 135) (Table S6, Supplemental Digital Content, http://links.lww.com/MD/N408). Similarly, AS was divided into an HR team (n = 249) and an LR team (n = 264) (Table S7, Supplemental Digital Content, http://links.lww.com/MD/N409). The K-MSC analysis suggested that the OS of the LR group was longer than that of the AS, TA, and VS (Fig. 2F–H). At the same time, the PFS of the LR group was longer than that of the AS group (Fig. 2I).

Next, we conducted risk curve, ROC curve, and PCA for AS, TS, and VS. Risk curve and survival analyses demonstrated that the higher the risk score, the higher the risk of death (Fig. 2J–L) and the higher the number of deaths (Fig. 2M–O). The risk heatmap showed that with increasing patient risk, the expression levels of AC003035.2, AC010273.3, AL450270.1, and LINC01571 increased, while the expression levels of AC010157.2, AC011444.3, AC092667.1, and AL645608.2 decreased (Fig. 2P–R). Subsequently, the AUC of ROC was applied to assess the prognostic ability of the DAlncRNAs. The 1-, 3-, and 5-year AUCs for AS were 0.916, 0.862, and 0.765, respectively (Fig. 2S). The 1-, 3-, and 5-year AUCs in the TS were 0.936, 0.934, and 0.860, respectively (Fig. 2T). The 1-, 3-, and 5-year AUCs in the VS were 0.908, 0.761 and 0.638, respectively (Fig. 2U). PCA was employed to visualize the differences between the 2 teams. The results showed that, compared with the models constructed using other sets (all genes, DAGs, and all DAlncRNAs), the model constructed using 8 DAlncRNAs had the most obvious discrimination between the HR and LR groups (Fig. 2V–Y).

### 3.3. The DAlncRNAs-based signature is an independently prognostic factor for LGG patients

**Figure 3.**
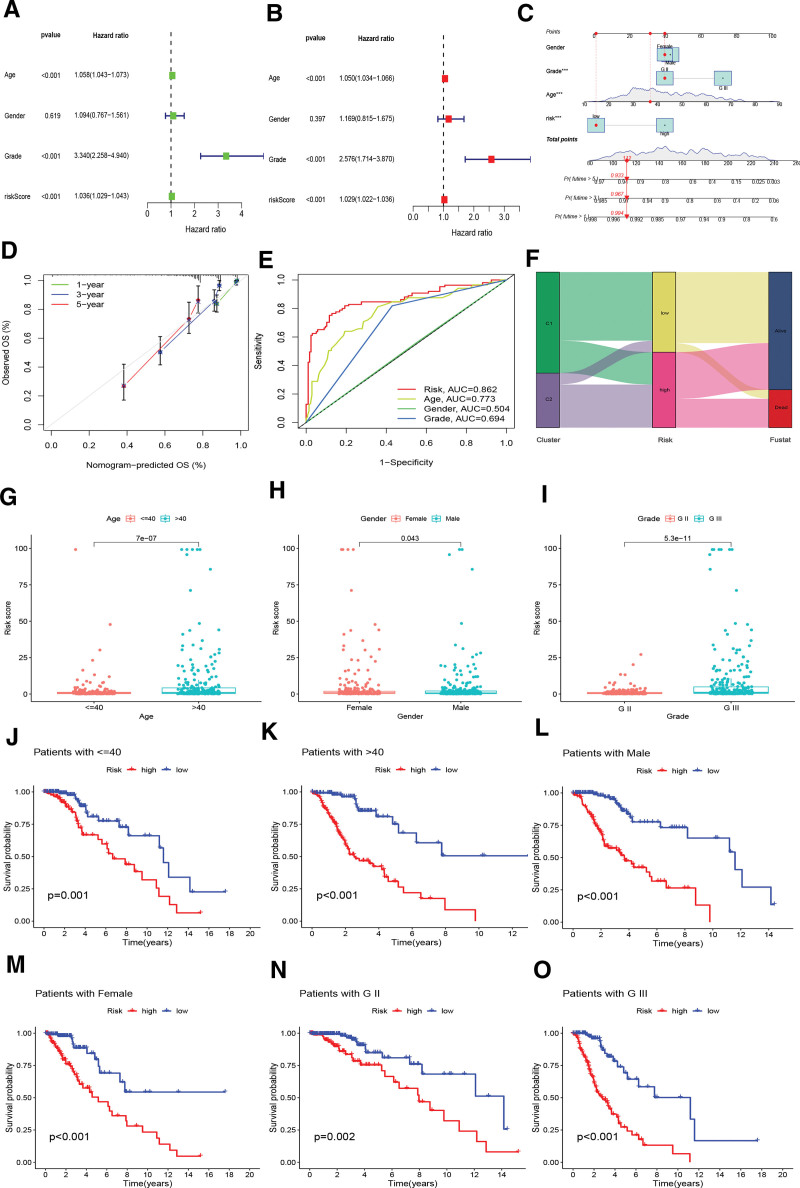
Analysis of independently prognostic factors. (A and B) Forest plots of clinical features and risk scores by univariate and multivariate Cox regression analysis. (C) A nomogram based on the risk score and clinical factors for predicting 1-, 3-, and 5-year survival in LGG patients. (D) Calibration of the nomogram. (E) ROC curves for different clinical features and risk score. (F) The Sankey map shows the link between subtypes of disulfidptosis, risk groups of disulfidptosis and survival states. (G–I) Differences in risk scores by age, sex, and grade. (J–O) Survival analysis of LGG patients ≤40 years (J), >40 years (K), male (L), female (M), grade WHO II (N), and grade WHO III (O). LGG = low-grade glioma, ROC = receiver operating characteristic.

Univariate and multivariate Cox regression analyses (Table S8, Supplemental Digital Content, http://links.lww.com/MD/N410) were performed to determine whether the DAlncRNAs-based signature was an independent prognostic element of LGG. The combined results revealed that age, tumor grade, and risk score were independent prognostic factors (Fig. 3A and B). Subsequently, to facilitate risk modeling to aid clinicians in decision-making, we utilized age, sex, grade, and risk score to create a nomogram for predicting the survival rate of patients with LGG (Fig. 3C). The calibration curve results suggested good consistency between the predicted and actual survival rates (Fig. 3D). The accuracy of the prognostic indicators was then evaluated using ROC curve analysis, which showed that risk scores predicted prognosis more accurately than age and grade (Fig. 3E). The Sankey diagram shows that the LR team was predominantly composed of the C1 subtype, with the majority of surviving patients coming from this team. In contrast, the patients who died were predominantly subtype C2, which constituted the HR team (Fig. 3F). We also found differences in the patient risk scores according to age, sex, and grade (Fig. 3G–I). To study the prognostic capability of the signature based on DAlncRNAs in LGG of different ages, grades and sexes, we performed survival analyses of LGG patients aged ≤ 40 years (Fig. 3J), >40 years (Fig. 3K), male (Fig. 3L), female (Fig. 3M), WHO grade II (Fig. 3N), and WHO grade III (Fig. 3O). The survival analysis results demonstrated that the prognosis of HR patients was noticeably worse than that of LR patients (Fig. 3G–L).

### 3.4. Risk model-based analysis of risk variance, functional enrichment, and the immunologic microenvironment of LGG cells

**Figure 4.**
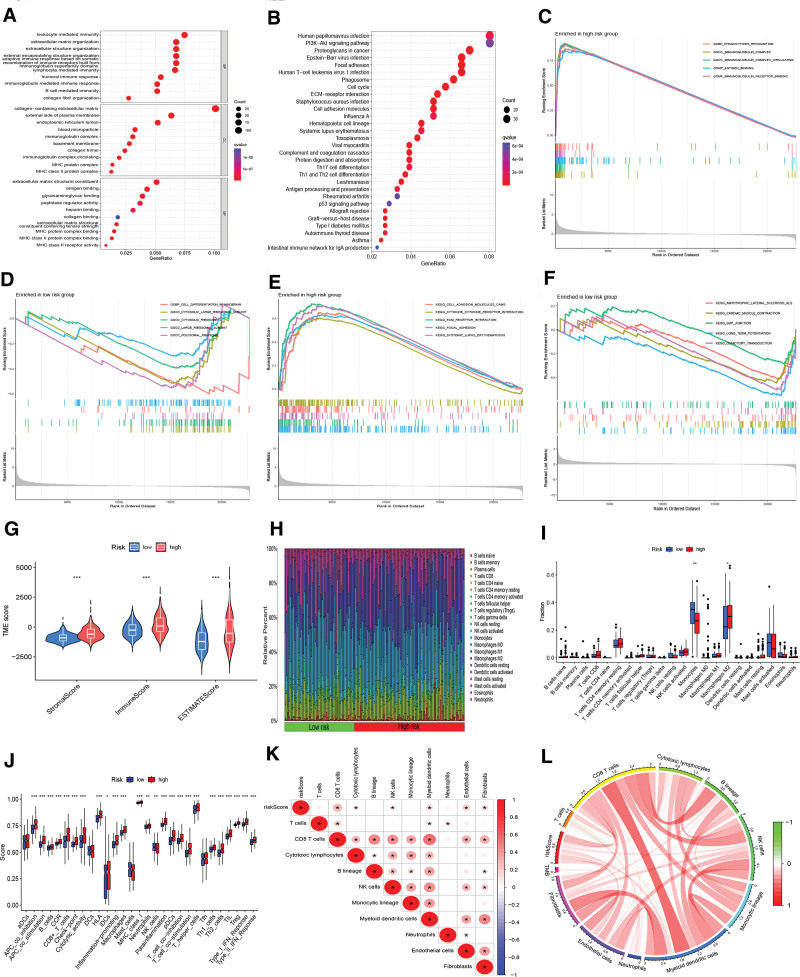
Functional enrichment, immune microenvironment and immune cell infiltration analysis. (A) GO analysis of RDEGs. (B) KEGG analysis of RDEGs. (C–F) GSEA of the HR and LR groups. (G) Differences in TME scores among different risk groups. (H) The proportion of infiltration of 22 tumor immune cells. (I) The box chart shows the difference in immune cell infiltration between the HR and LR group. (J) Box diagrams for analysis of immune-related functional differences between the HR and LR groups. (K) The heatmap of correlation between immune cells and risk scores. (L) A circle plot of relevance between immune cells. GO = Gene Ontology, GSEA = gene set enrichment analysis; HR = high-risk, KEGG, Kyoto Encyclopedia of Genes and Genomes, LR = low-risk , RDEGs = risk differentially expressed genes.

According to the differences in gene expression between the HR and LR groups, 1288 RDEGs were identified (Table S9, Supplemental Digital Content, http://links.lww.com/MD/N411). GO analysis indicated that RDEGs were mainly involved in immune-related functions, such as leukocyte-mediated immunity and humoral immune responses (Fig. 4A). KEGG analysis suggested that these genes were mainly associated with pathways such as human papillomavirus infection, the PI3K-Akt signaling pathway, proteoglycans in cancer, and phagolysosomes (Fig. 4B). GSEA revealed that the HR group was mainly enriched in 5 GO functions, such as GOBP_PHAGOCYTOSIS_RECOGNITION (Fig. 4C), and 5 KEGG pathways, such as KEGG_SYSTEMIC_LUPUS_ERYTHEMATOSUS (Fig. 4E). In contrast, the LR group was mainly enriched in 5 GO functions, such as GOBP_CELL_DIFFERENTIATION_IN_HINDBRAIN (Fig. 4D), and 5 KEGG pathways, such as KEGG_OLFACTORY_TRANSDUCTION (Fig. 4F).

Next, we explored the link between the tumor microenvironment (TME) and immune-related cells in the HR and LR groups. The estimated results showed that the TME scores differed between the 2 groups, and the HR group contained higher levels of stromal and immune cells (Fig. 4G). Immune infiltration analysis suggested higher monocyte infiltration in the LR group, whereas M2 macrophage infiltration was more prominent in the HR group (Fig. 4H, I). Differential analysis of immune-related functions indicated that there were notable differences in immune functions between the LR and HR groups, and 24 functions, including APC_co_inhibition, were more active in the HR group (Fig. 4J). CD8+ T cells, cytotoxic lymphocytes, NK cells, myeloid dendritic cells, endothelial cells, and fibroblasts were positively correlated with risk score (Fig. 4K). The correlation between immune cells showed that CD8+ T cells had a synergistic effect with a variety of cells, such as fibroblasts (Fig. 4L).

### 3.5. Expression and mutation of DAGs in LGG and gene mutation analysis on the basis of the prognostic signature

**Figure 5.**
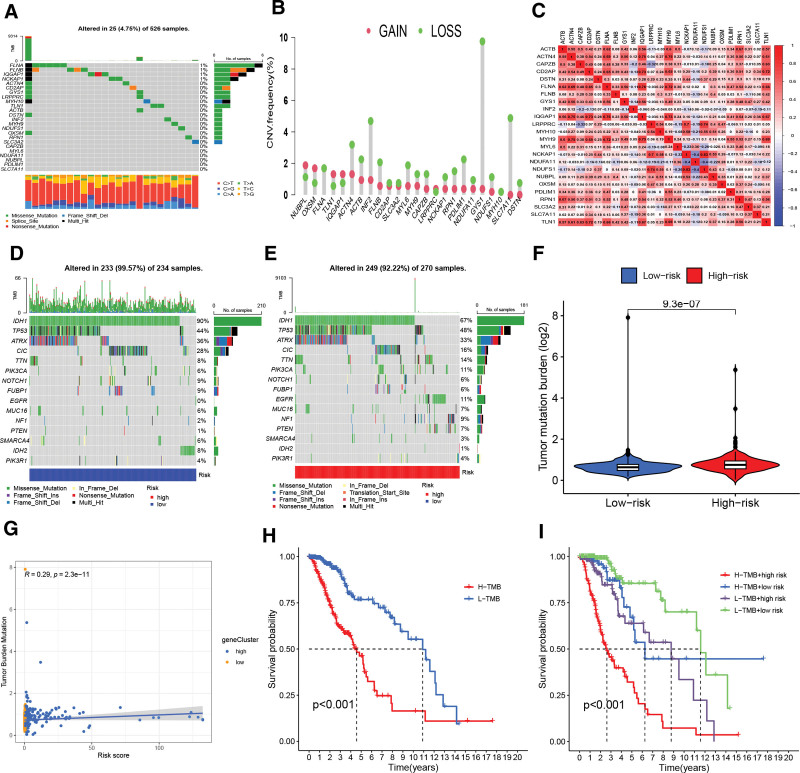
Genetic alterations and TMB analysis. Figure 2 genetic alterations of DAGs in LGG. (A) The mutational rates of the 24 DAGs in 526 LGG samples. (B) The copy number changes in 24 DAGs. (C) Correlation heatmap illustrating the correlation coefficients of the 24 DAGs. (D and E) The waterfall plots show the top 15 genes with the maximum mutation frequency in the LR (D) and HR teams (E). (F) TMB scores of the LR and HR teams. (G) Relevance between the risk score and TMB. (H) K-MSC showed the OS of L-TMB and H-TMB group. (I) K-MSC showed the OS of the 4 subgroups. DAGs = disulfidptosis-associated genes, HR = high-risk, K-MSC = Kaplan-Meier survival curves, LGG = low-grade glioma, LR = low-risk, TMB = tumor mutation burden.

To probe the role of DAGs in LGG, we studied the mutational rates of DAGs in LGG, and the results revealed an extraordinarily low percentage of mutations in the DAGs (Fig. 5A). Of the 526 LGG samples in TCGA cohort, only 25 (4.75%) were regulated by DAG mutations. The mutation rates of FLNA, FLNB, IQGAP1, and NCKAP1 were higher, whereas CAPZB and other genes did not appear in the samples. Among the 24 genes, 5 genes, including NUBPL, showed an increased frequency of CNV amplification, and 19 genes, including OXSM, exhibited an increased frequency of CNV deletion (Fig. 5B). Gene association analysis demonstrated that most of the 24 DAGs, including FLNA and IQGAP1, showed strong synergistic effects (Fig. 5C).

Meanwhile, we compared the upper 15 genes with the maximum frequency of changes in the HR and LR teams. Among these, IDH1, TP53, and ATRX were ranked high. The mutation frequencies of CIC, IDH1, NOTCH1, ATRX, FUBP1, SMARCA4, and IDH2 were dominant in the LR group compared to the HR group, whereas TP53, TTN, PIK3CA, EGFR, MUC16, NF1, and PTEN were at a disadvantage (Fig. 5D and E). We then analyzed TMB and found remarkable differences between the 2 groups, with a higher TMB in the HR group (Fig. 5F). There was a positive relationship between TMB and the risk score (Fig. 5G). Thus, the DAlncRNAs-based prognostic signature was linked to TMB. The K-MSC demonstrated that the prognosis of the H-TMB group was worse than that of the L-TMB group (Fig. 5H), suggesting that TMB might be an implicating factor in the worse prognosis of patients with LGG. Therefore, we classified the patients into 4 subgroups (H-TMB + HR, H-TMB + LR, L-TMB + HR, and L-TMB + LR) and carried out survival analysis to facilitate the understanding of the comprehensive impact of risk scores and TMB on patient survival. The outcomes suggested that the prognosis was the worst in the H-TMB + HR team and the best in the L-TMB + LR team (Fig. 5I).

### 3.6. Immunotherapy and chemotherapy predictions

**Figure 6.**
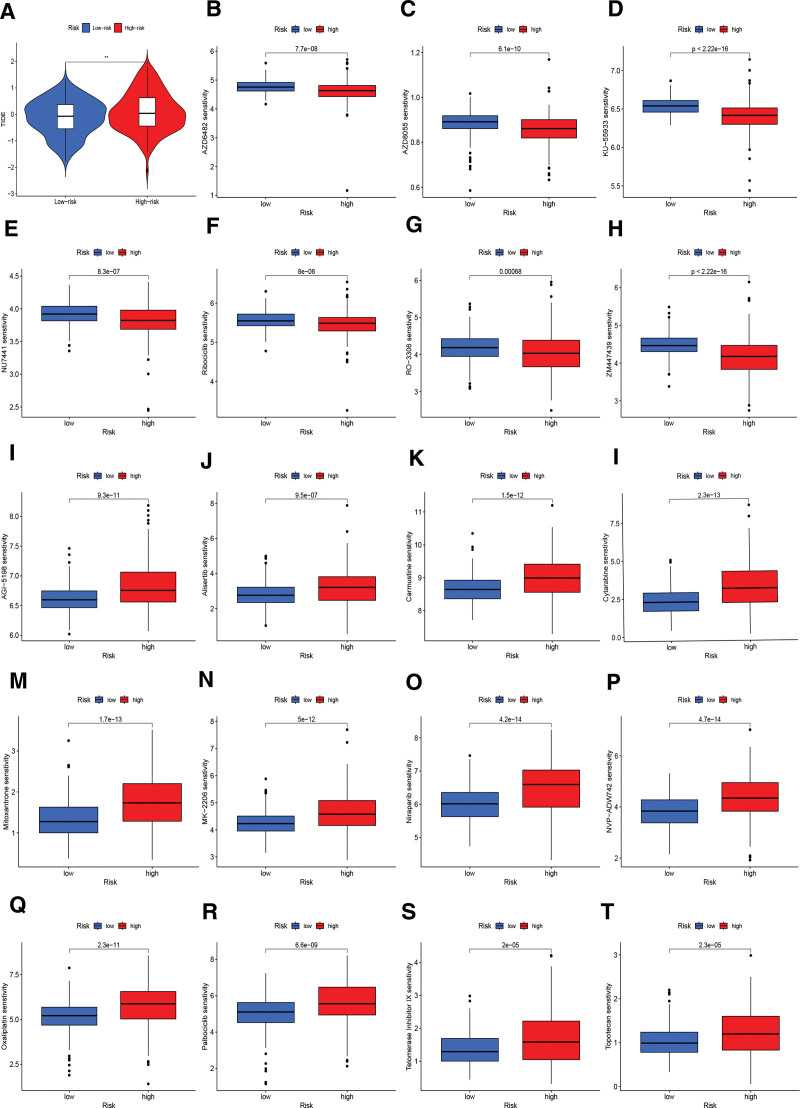
Immunotherapy and chemotherapy in both risk groups. (A) Differences in tide scores across the HR and LR teams. (B–T) Sensitivity differences of 19 drugs in different risk groups. HR = high-risk, LR = low-risk.

To investigate the influence of risk scores on immunotherapy, we used the TIDE score to assess the ability of LGG immune evasion to predict the immune checkpoint blocking response. The violin plot indicated that the LR team patients were more probably to respond to immunotherapy (Fig. 6A). Simultaneously, 19 sensitive drugs were identified in the HR and LR groups. The drug sensitivity results exhibited that AZD6482 (PI3K β inhibitor), KU-55933 (ATM inhibitor), AZD8055 (mTOR inhibitor), ribociclib (CDK46 inhibitor), RO-3306 (CDK1 inhibitor) NU7441, (DNA-PK inhibitor), and ZM447439 (aurora inhibitor) showed high sensitivity in the LR team (Fig. 6B–H). In the HR team, AGI-5198 (R132H-IDH1 inhibitor), alisertib, carmustine, cytarabine, mitoxantrone, MK-2206 (Akt inhibitor), niraparib, NVP-ADW742 (IGF-1 receptor kinase inhibitor), oxaliplatin, palbociclib, telomerase inhibitor IX, and topotecan presented high sensitivity (Fig. 6I–T).

## 4. Discussion

Surgical removal of tumors, adjuvant radiotherapy, and chemotherapy are conventional treatments for LGG. However, these treatments cannot be cured, and the survival rate of patients remains limited; therefore, more creative and effective interventions are needed. Previous studies have suggested that the aggregation of S–S bonds in the mitochondria modulates tumor advancement.^[22]^ Disulfidptosis is a type of cell death triggered by S–S bond stress, which is characterized by the collapse of cytoskeletal proteins and F-actin due to the intracellular accumulation of disulfides.^[9]^ This discovery could eventually offer new avenues for cancer therapy. In the present work, we revealed 2 disulfidptosis of LGG isoforms with distinct prognoses and immune characteristics, and established a new LGG prognosis model based on DAlncRNAs.

NMF is considered a method that is helpful in realizing precise medical treatment and has been actively used for gene expression, mutation characteristics, multigroup, single cell, and meta-analysis in the field of oncology.^[23]^ In our study, the NMF algorithm was applied to categorize patients with LGG into 2 subgroups (C1 and C2) associated with disulfidptosis according to the expression of DAlncRNAs. C2 patients with large changes in immune cells have a poor prognosis. Therefore, disulfidptosis-related regulatory factors not only cause alterations in the immune system but also in the immune microenvironment and related functions in patients with LGG.

In the current research, we identified 8 DAlncRNAs that are linked to the prognosis of LGG. Among them, AC003035.2, AC010273.3, AL450270.1, and LINC01571 were HR lncRNAs that were positively linked to the risk score, whereas AC010157.2, AC011444.3, AC092667.1, and AL645608.2 were LR lncRNAs that were negatively linked to the risk score. Noteworthily, it has been reported that AL450270.1 is an immune-associated lncRNA that may be linked to the prognosis of patients with thymic epithelial tumors,^[24]^ while AL645608.2 is highly related to the clinical prognosis of patients with acute myeloid leukemia.^[25]^ Our research developed the first DAlncRNAs prognostic signature for LGG. ROC curve analysis, risk curve analysis, and PCA also verified the precision of the signature prediction. One-, three-, and five years corresponded to AUC values of 0.916, 0.862, and 0.765, respectively. Therefore, the signature based on the 8 DAlncRNAs had a promising prognostic ability. Univariate and multivariate Cox regression analyses confirmed that the DRlncRNAs-based risk model was an independent prognostic factor for patients with LGG. Risk scores predicted the prognosis with better accuracy than age and grade. In addition, the nomogram related to disulfidptosis was highly valuable in predicting survival time in patients with LGG.

GO and KEGG analyses illustrated that the RDEGs were mainly implicated in immune-related functions, infection, and cancer-related pathways. GSEA results also revealed that the genes in the HR group were mostly involved in immune-associated functions, cancer, and immune-related pathways. Therefore, these functions and pathways may contribute to the formation of the tumor immune microenvironment in LGG. TME analysis suggested that the HR group contained higher levels of stromal and immune cells. Moreover, M2 macrophage infiltration was prominent in the HR team. Foregoing studies have implicated M2 macrophages in the remarkable malignant features of LGG.^[26]^ The number of M2 macrophages can be applied to assess tumor grade and prognosis in glioma patients.^[27]^ Besides, in the HR group, the immune function was more active, the patients were mainly composed of subtype C2, and the prognosis was worse. Therefore, M2 macrophages may be negatively linked to LGG survival. However, it would be worthwhile to further investigate the mechanism of M2 macrophages in LGG.

Adjacently, we determined the 3 most mutated genes in the HR and LR teams, namely IDH1, TP53, and ATRX. IDH is an enzyme with 3 subtypes (IDH1, IDH2, and IDH3). IDH mutations are observed in approximately 80% of WHO grade II and III gliomas, and LGG with such mutations is more likely to develop into secondary GBM.^[28,29]^ In this study, the IDH1 mutation rates were as high as 90% and 67% in the LR and HR teams, respectively. TMB was predominant in the HR team, the risk score was positively linked to TMB, and the prognosis of the H-TMB group was worse than that of the L-TMB group, suggesting that TMB might be an implicating factor in worse prognosis in patients with LGG. Meanwhile, the prognosis was worst in the H-TMB + HR team and best in the L-TMB + LR team, which supported the validity of the signature. In summary, gene mutation analysis on the basis of the prognostic signature further bolsters the prognostic implication of the disulfidptosis-associated prognostic signature (DAPS) and gives a precious reference for investigating the potential molecular pathogenesis of LGG.

Subsequently, we used the TIDE algorithm to investigate the immunotherapeutic value of disulfidptosis-related features to predict immunotherapeutic response. In the present study, the HR group had a remarkably higher TIDE score and a lower effective immune response rate, suggesting that LR patients may be more appropriate for immunotherapy. The addition of procarbazine, lomustine, and vincristine to radiotherapy is a cost-effective therapy strategy for HR patients with LGG. Temozolomide also benefits HR LGG. Moreover, drugs targeting IDH1 mutations are currently under research or development, including IDH inhibitors, poly ADP ribose polymerase, and demethylated drugs.^[2]^ Drug sensitivity analysis of HR and LR groups can provide a reference for LGG treatment.

In short, we highlighted 2 subtypes of LGG associated with disulfidptosis, which have distinct prognostic and immune characteristics. Furthermore, we have developed a new DAPS, which not only provides a valuable reference for investigating the potential molecular pathogenesis of LGG but also effectively predicts the prognosis of LGG patients. This can assist clinicians in making clinical decisions and offer new perspectives on individualized treatment for LGG. Besides, it is of concern that there is also a lack of disulfidptosis-related inhibitors or biomarkers for in vivo analysis. The main regulatory pathways involved in disulfidptosis are the cystine uptake and glucose metabolism pathways, of which SLC7A11 is a key factor.^[9]^ Further studies are needed to determine whether the hypersensitivity to glucose transporter protein inhibition observed in tumors with high SLC7A11 involves other mechanisms.^[30]^ In addition, further exploration and validation of disulfidptosis-targeting strategies in both preclinical and clinical settings may usher in a new era of precision medicine for SLC7A11-high cancers or other related diseases.^[30,31]^

No doubt, this study has some limitations. First, we only used LGG data from TCGA for our study; therefore, more LGG data are necessary to test the practicability of the signature and the reliability of immunotherapy and chemotherapy predictions. Secondly, the present study was based on bioinformatics analysis, and more cellular and animal experiments are necessary to validate the expression and specific biological functions of the 8 DAlncRNAs identified in LGG.

## 5. Conclusions

Our study, based on DAlncRNAs, highlights 2 disulfidptosis-associated LGG subtypes with different prognostic and immune characteristics and creates a novel DAPS, which may inform the classification, prognosis, molecular pathogenesis, and therapeutic strategies for patients with LGG.

## Acknowledgments

We appreciate all the researchers in this work.

## Author contributions

**Conceptualization:** Xiaohong Qin, Zhibiao Chen, Liquan Wu, Rui Ding.

**Data curation:** Xiaohong Qin, Zhibiao Chen, Liquan Wu, Rui Ding.

**Formal analysis:** Xiaohong Qin, Rui Ding.

**Funding acquisition:** Zhibiao Chen, Rui Ding.

**Investigation:** Xiaohong Qin.

**Methodology:** Xiaohong Qin, Rui Ding.

**Project administration:** Zhibiao Chen, Liquan Wu, Rui Ding.

**Resources:** Xiaohong Qin.

**Software:** Xiaohong Qin.

**Supervision:** Xiaohong Qin, Rui Ding.

**Validation:** Xiaohong Qin, Rui Ding.

**Visualization:** Xiaohong Qin.

**Writing – original draft:** Xiaohong Qin.

**Writing – review & editing:** Xiaohong Qin, Zhibiao Chen, Liquan Wu, Rui Ding.

## Supplementary Material

**Figure:**