# Supplementary material for: A disulfidptosis-associated long noncoding RNA signature to predict low-grade glioma classification, prognosis, tumor microenvironment, and therapy regimens: Observational study
Source: Medicine (Baltimore). 2024 Aug 23;103(34):e39316. doi: 10.1097/MD.0000000000039316 (PMC11346906; doi:10.1097/MD.0000000000039316)
Supplement: Supplementary file 3 [file medi-103-e39316-s003.docx]

**Table S3** Statistical analysis of clinical groups

| **Characteristic** | **Type** | **Total** | **Test** | **Train** | ***Χ^2^*** | **P value** |
| --- | --- | --- | --- | --- | --- | --- |
| Age | <=40 | 253(49.32%) | 121(47.27%) | 132(51.36%) | 0.8609 | 0.4012 |
|  | >40 | 260(50.68%) | 135(52.73%) | 125(48.64%) |  |  |
| Gender | Female | 228(44.44%) | 110(42.97%) | 118(45.91%) | 0.4507 | 0.5602 |
|  | Male | 285(55.56%) | 146(57.03%) | 139(54.09%) |  |  |
| Grade | G2 | 247(48.15%) | 125(48.83%) | 122(47.47%) | 1.0681 | 0.8596 |
|  | G3 | 265(51.66%) | 131(51.17%) | 134(52.14%) |  |  |
|  | unknow | 1(0.19%) | 0(0%) | 1(0.39%) |  |  |
