# Supplementary material for: A disulfidptosis-associated long noncoding RNA signature to predict low-grade glioma classification, prognosis, tumor microenvironment, and therapy regimens: Observational study
Source: Medicine (Baltimore). 2024 Aug 23;103(34):e39316. doi: 10.1097/MD.0000000000039316 (PMC11346906; doi:10.1097/MD.0000000000039316)
Supplement: Supplementary file 4 [file medi-103-e39316-s005.docx]

**Table S4** The eight prognostic DRlncRNAs

| **lncRNA** | **coef** | **HR** | **HR.95L** | **HR.95H** | **p value** |
| --- | --- | --- | --- | --- | --- |
| AC003035.2 | 0.5590 | 3.1688 | 2.3071 | 4.3523 | 1.06E-12 |
| AC010157.2 | -0.7938 | 0.3945 | 0.2527 | 0.6157 | 4.23E-05 |
| AC010273.3 | 0.6228 | 2.9003 | 2.1826 | 3.8539 | 2.11E-13 |
| AC011444.3 | -0.3397 | 0.4106 | 0.2827 | 0.5963 | 2.93E-06 |
| AC092667.1 | -0.9616 | 0.1733 | 0.0716 | 0.4194 | 1.02E-04 |
| AL450270.1 | 0.2945 | 2.5474 | 1.9429 | 3.3399 | 1.32E-11 |
| AL645608.2 | -0.4732 | 0.4545 | 0.3256 | 0.6343 | 3.56E-06 |
| LINC01571 | 0.6249 | 3.5264 | 2.3987 | 5.1841 | 1.45E-10 |
