# Supplementary material for: A disulfidptosis-associated long noncoding RNA signature to predict low-grade glioma classification, prognosis, tumor microenvironment, and therapy regimens: Observational study
Source: Medicine (Baltimore). 2024 Aug 23;103(34):e39316. doi: 10.1097/MD.0000000000039316 (PMC11346906; doi:10.1097/MD.0000000000039316)
Supplement: Supplementary file 8 [file medi-103-e39316-s009.docx]

**Table S8** The results of univariate and multivariate Cox regression analysis

| **Charac-teristic** | **uniCox** | | | |  | **multiCox** | | | |
| --- | --- | --- | --- | --- | --- | --- | --- | --- | --- |
|  | **HR** | **HR.95L** | **HR.95H** | **P value** |  | **HR** | **HR.95L** | **HR.95H** | **P value** |
| Age | 1.0583 | 1.0433 | 1.0734 | 6.86E-15 |  | 1.0503 | 1.0343 | 1.0665 | 3.26E-10 |
| Gender | 1.0945 | 0.7671 | 1.5614 | 6.19E-01 |  | 1.1686 | 0.8152 | 1.6751 | 3.97E-01 |
| Grade | 3.3397 | 2.2577 | 4.9403 | 1.57E-09 |  | 2.5759 | 1.7144 | 3.8704 | 5.24E-06 |
| Risk Score | 1.0361 | 1.0293 | 1.0429 | 5.44E-26 |  | 1.0288 | 1.0216 | 1.0360 | 1.49E-15 |
